# Circular RNA circ‐TSPAN4 promotes lung adenocarcinoma metastasis by upregulating ZEB1 via sponging miR‐665

**DOI:** 10.1002/mgg3.2165

**Published:** 2023-04-12

Ying, X., Zhu, J., and Zhang, Y. “Circular RNA circ‐TSPAN4 promotes lung adenocarcinoma metastasis by upregulating ZEB1 via sponging miR‐665”. *Molecular Genetics & Genomic Medicine*. 2019, 7(12), e991. https://doi.org/10.1002/mgg3.991.

The above article, published online on 01 October 2019 in Wiley Online Library (https://onlinelibrary.wiley.com), has been retracted by agreement between the authors, the journal Editor‐in‐Chief Dr. Suzanne Hart, and John Wiley & Sons. The retraction has been agreed due to concerns raised by the authors regarding problems with the statistics and also concerns raised by a third party about image manipulation in Figure 2. The journal team have investigated and determined that these concerns undermine the reliability of the data presented and the article‘s conclusions.

